# Molecular helices as electron acceptors in high-performance bulk heterojunction solar cells

**DOI:** 10.1038/ncomms9242

**Published:** 2015-09-18

**Authors:** Yu Zhong, M. Tuan Trinh, Rongsheng Chen, Geoffrey E. Purdum, Petr P. Khlyabich, Melda Sezen, Seokjoon Oh, Haiming Zhu, Brandon Fowler, Boyuan Zhang, Wei Wang, Chang-Yong Nam, Matthew Y. Sfeir, Charles T. Black, Michael L. Steigerwald, Yueh-Lin Loo, Fay Ng, X.-Y. Zhu, Colin Nuckolls

**Affiliations:** 1 Department of Chemistry, Columbia University, 3000 Broadway, Havemeyer Hall, MC3130, New York, New York 10027, USA; 2 School of Chemical Engineering and Technology, Wuhan University of Science and Technology, Wuhan 430081, China; 3 Department of Chemical and Biological Engineering, Princeton University, Princeton, New Jersey 08544, USA; 4 Center for Functional Nanomaterials, Brookhaven National Laboratory, Upton, New York, New York 11973, USA

## Abstract

Despite numerous organic semiconducting materials synthesized for organic photovoltaics in the past decade, fullerenes are widely used as electron acceptors in highly efficient bulk-heterojunction solar cells. None of the non-fullerene bulk heterojunction solar cells have achieved efficiencies as high as fullerene-based solar cells. Design principles for fullerene-free acceptors remain unclear in the field. Here we report examples of helical molecular semiconductors as electron acceptors that are on par with fullerene derivatives in efficient solar cells. We achieved an 8.3% power conversion efficiency in a solar cell, which is a record high for non-fullerene bulk heterojunctions. Femtosecond transient absorption spectroscopy revealed both electron and hole transfer processes at the donor−acceptor interfaces. Atomic force microscopy reveals a mesh-like network of acceptors with pores that are tens of nanometres in diameter for efficient exciton separation and charge transport. This study describes a new motif for designing highly efficient acceptors for organic solar cells.

## Abstract (web summary)

In organic photovoltaics, the best-performing devices are often based on fullerene derivatives as the electron acceptor counterpart. Here, the authors present new molecular electron acceptors with a helical structure and achieve 8.3% power conversion efficiency.

## Graphical abstract

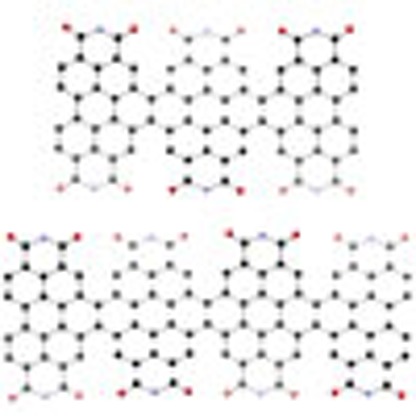

In the past decade, the record power conversion efficiency (PCE) of organic bulk heterojunction (BHJ) solar cells has reached over 10% for single junction cells^1^,^2^ and more than 12% for tandem cells^3^, mainly by synthesizing and incorporating new electron donors^4^,^5^, optimizing film morphology^1^,^6^, developing interfacial layers^7^,^8^,^9^ and designing new device structures^10^,^11^,^12^. One lagging area is the development of new electron acceptors for organic solar cells. To date, highly efficient BHJ solar cells almost exclusively use fullerene derivatives, such as [6,6]-phenyl-C_61/71_-butyric acid methyl ester (PC_61_BM and PC_71_BM), as electron acceptors^13^. Theoretical analysis suggests that the superiority of fullerene over non-fullerene electron acceptors is in the charge separation^14^. Recently, several studies, including our own, have reported solution-processed BHJ devices using non-fullerene acceptors that have achieved PCEs of 6%–7% (refs 15, 16, 17, 18, 19). Continued progress will require the design, synthesis and testing of new motifs for electron acceptors.

We herein use two helical *n*-type molecules that differ in their lengths as highly efficient electron acceptors. These fullerene-free BHJ solar cells achieve PCEs of 8.3%, with high short-circuit current density (15.2 mA cm^−2^) and fill factors (FFs) as large as 68%. The film morphology of the mixed donor/acceptor active layer is a mesh-like network of acceptors with pores that are tens of nanometres in diameter. Transient absorption (TA) spectroscopy reveals that excitons generated in both the donor and acceptor phases contribute to the photocurrent by effective hole and electron transfer at the interface between donor and acceptor.

## Results

### Molecule design and characterization

**Figure 1:**
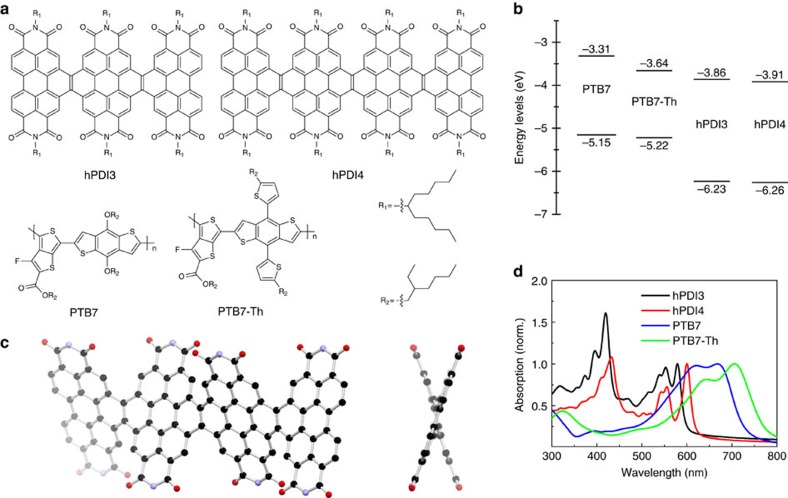
Molecular structures of helical perylene diimide oligomers and polymers. (**a**) Chemical structures of hPDI3, hPDI4, PTB7 and PTB7-Th. (**b**) Schematic of the energy levels of hPDI3, hPDI4, PTB7 and PTB7-Th. Energy levels of PTB7 and PTB7-Th were adopted from ref. 7 and ref. 5, respectively. Energy levels of hPDI3 and hPDI4 were adopted from ref. 21. (**c**) Molecular model of hPDI4 in a waggling conformation from DFT calculations. Hydrogens and alkyl side chains have been removed for clarity. Black, carbon; red, oxygen; blue, nitrogen. (**d**) Film absorption spectra of hPDI3, hPDI4, PTB7 and PTB7-Th normalized at their low-energy *λ*_max_.

The two helices have different lengths and are constructed by fusing either three or four perylene diimide (PDI) units together with a two-carbon bridge (hPDI3 and hPDI4 in Fig. 1a)^20^. Recently, we found that a shorter version of helical molecules was useful in BHJ solar cells^16^. Similar to the shorter one, the longer acceptors explored here also have relatively high electron mobilities (0.04–0.05 cm^2^ V^−1^ s^−1^ in thin film transistors), good electron-accepting ability and lowest unoccupied molecular orbital (LUMO) levels (approximately −3.9 eV) that are appropriately matched to those of commercially available electron-donating polymers (Fig. 1b)^20^. Similar to prior non-fullerene electron acceptors that have been moderately successful in solar cells^15^,^21^,^22^, these molecules possess nonplanar molecular structures due to the steric congestion in the cove areas defined by the junction point between the PDIs. There are several iso-energetic conformations within these oligomers because of the inversion of the helicity at each of these junctions (Fig. 1c, Supplementary Fig. 1 and Supplementary Note 1). Because of the nonplanar structure and the associated conformational dynamics, these molecules do not aggregate strongly in thin films^23^. Both of the molecules absorb light strongly in the wavelength range from 350 to 600 nm (Fig. 1d) with a maximum molar extinction coefficient of 1.5 × 10^5^ M^−1^ cm^−1^ for hPDI3 and 1.8 × 10^5^ M^−1^ cm^−1^ for hPDI4 (see Supplementary Fig. 2a.). The strong light absorption, the lack of aggregation and the propensity of the isolated linear structure to form networks^24^ indicate that these two molecules have the potential to outperform PDI monomers and dimers in BHJ solar cells.

### Solar cell characterization

Our device demonstrations using the compounds hPDI3 and hPDI4 as electron acceptors are based on the commercially available low bandgap semiconducting polymer donors polythieno[3,4-b]-thiophene-co-benzodithiophene (PTB7)^4^ and poly[4,8-bis(5-(2-ethylhexyl)thiophen-2-yl)benzo[1,2-b;4,5-b′]dithiophene-2,6-diyl-alt-(4-(2-ethylhexyl)-3-fluorothieno[3,4-b]thiophene)-2-carboxylate-2,6-diyl] (PTB7-Th)^5^ (shown in Fig. 1a). The absorption bands of PTB7 and PTB7-Th are red shifted relative to those of hPDI3 and hPDI4 (Fig. 1d). Films of hPDI3 or hPDI4 blended with PTB7 or PTB7-Th show broad, intense absorptions spanning the wavelength range from 350 to 800 nm (Supplementary Fig. 2b).

**Figure 2:**
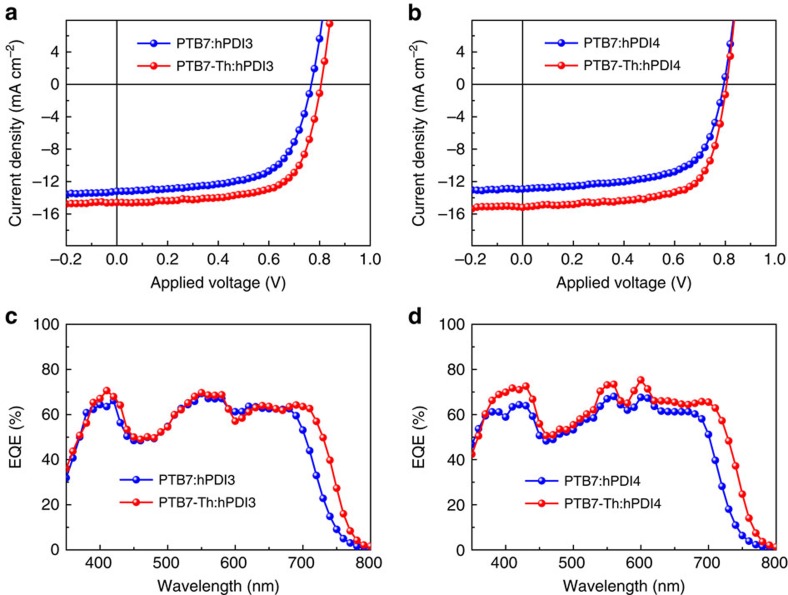
Device performance of best solar cells based on molecular helices. *J*–*V* curves for (**a**) PTB7:hPDI3 and PTB7-Th:hPDI3 solar cells, (**b**) PTB7:hPDI4 and PTB7-Th:hPDI4 solar cells under optimized conditions and simulated AM 1.5G irradiation (100 mW cm^−2^). EQE spectra of (**c**) PTB7:hPDI3, PTB7-Th:hPDI3 and (**d**) PTB7:hPDI4, PTB7-Th:hPDI4 solar cells under optimized conditions.

For each combination of blended polymer and helical molecule device active layer, we varied the ratio of donor and acceptor, and optimized the film morphology with the solvent additive diiodooctane (DIO)^6^ (see details about device optimization process in Supplementary Figs 3–6 and Supplementary Tables 1–4). Our devices were fabricated in an inverted configuration, using ZnO as the electron transport layer and MoO_3_ as the hole transport layer^13^. For devices based on acceptor hPDI3, we obtain optimal device performance with donor:acceptor mass ratio of 1:1.5, with 1% DIO additive (v/v). Typical current density−voltage (*J−V*) curves for PTB7:hPDI3 and PTB7-Th:hPDI3 solar cells are shown in Fig. 2a. The solar cell parameters are listed in Table 1. PTB7-Th:hPDI3 exhibits a larger *J*_sc_ of 14.5 mA cm^−2^ as compared with 13.2 mA cm^−2^ for PTB7:hPDI3, owing to the red-shifted absorption of PTB7-Th relative to PTB7. Although the open-circuit voltage (*V*_oc_) is comparable at 0.77–0.81 eV, the FF increases from 63% for PTB7:hPDI3 to 67% for PTB7-Th:hPDI3. Overall, devices comprised of PTB7-Th:hPDI3 outperform those that comprise PTB7:hPDI3, exhibiting a maximal PCE of 7.9% for PTB7-Th:hPDI3 as compared with 6.4% for PTB7:hPDI3.

For solar cells based on the electron acceptor hPDI4, we achieved optimal device performance with donor-to-acceptor mass ratio of 1:1 and 1% DIO additive (v/v). Typical current density–voltage curves are shown in Fig. 2b. Optimized devices show similar performance to those based on hPDI3, with PTB7:hPDI4 and PTB7-Th:hPDI4 solar cells reaching maximum PCE of 6.5% and 8.3%, respectively (device characteristics summarized in Table 1). Device performance of a PTB7-Th:hPDI4 solar cell has been independently certified by Newport Corporation, as shown in Table 1 and Supplementary Fig. 7. These device characteristics are similar to those reported in fullerene-based cells (PTB7-Th:PC_71_BM) with the same interfacial layers (ZnO and MoO_3_)^2^,^5^,^25^,^26^ and represent record highs for non-fullerene BHJs, with the highest non-fullerene BHJ having a PCE of 6.8% (refs 15, 16, 17, 18). These molecules are the first example of electron acceptors that are on par with fullerene derivatives in efficient BHJ solar cells with a PCE >8% and provide a new route to exploring improvements to BHJ device performance.

Previously reported solar cells with non-fullerene acceptors typically have *J*_sc_ <14 mA cm^−2^ and FF values below 60% (refs 15, 16, 17, 18), which may be indicators of sub-optimal charge-carrier collection. The improved *J*_sc_ and FF values in devices containing helical PDIs may be due to the efficient exciton generation, separation and carrier transport. Plots of the photocurrent density (*J*_ph_) versus effective voltage (*V*_eff_) yield information about exciton generation rates and the charge collection probabilities *P*(*E*,*T*) at *J*_sc_ conditions (Supplementary Fig. 8). Here, *V*_eff_ is defined as *V*_eff_=*V*_0_−*V*_a_, where *V*_0_ is the voltage where *J*_ph_ equals zero and *V*_a_ is the applied bias voltage. For both hPDI3-based and hPDI4-based devices, *J*_ph_ reaches the saturation current density at a relatively low *V*_eff_ (1.8 V). The PTB7-Th-based devices have slightly higher saturation current density than the PTB7-based device, consistent with the trend of *J*_sc_ values, and is attributed to its broader spectral response (Supplementary Table 5). Under *J*_sc_ conditions, the *P*(*E*,*T*) is 94% for PTB7:hPDI3 and as high as 97% for PTB7:hPDI4, PTB7-Th:hPDI3 and PTB7-Th:hPDI4 devices. From the light-intensity-dependent current density measurement, we find a near-unity exponent *α* in the expression of *J*_sc_ versus *I*^*α*^, where *I* is incident light intensity (see Supplementary Figs 9–16). This data also suggests that bimolecular recombination and space–charge effect are suppressed at *J*_sc_ conditions^27^.

We measure the electron and hole mobilities in the optimal blend films by the space–charge-limited current method^28^. For PTB7-Th:hPDI3 blended film, the hole and electron mobilities are (1.0±0.1) × 10^−4^ and (1.5±0.1) × 10^−4^ cm^2^ V^−1^ s^−1^, respectively. For PTB7-Th:hPDI4 blended film, the hole and electron mobilities are (1.2±0.1) × 10^−4^ and (1.5±0.3) × 10^−5^ cm^2^ V^−1^ s^−1^, respectively (see Supplementary Fig. 17 and Supplementary Note 2). The lower electron mobility in PTB7-Th:hPDI4 compared with that of PTB7-Th:hPDI3 is probably due to reduced mass ratio of the electron acceptor in the blended film (50% in PTB7-Th:hPDI4 versus 60% in PTB7-Th:hPDI3). PTB7-Th:hPDI4 blended film exhibits similar electron mobility to fullerene-based blended film^29^, while PTB7-Th:hPDI3 shows even more balanced electron and hole mobility.

Figure 2c,d display the external quantum efficiency (EQE) spectra for each of the devices. These devices show broad and strong photoresponse from 350 to 800 nm. Compared with the PTB7-based solar cells, the PTB7-Th solar cells show a significant increase in the photoresponse between 700 and 800 nm due to the red-shifted absorption of PTB7-Th. The integrated *J*_sc_ values for PTB7:hPDI3, PTB7:hPDI4, PTB7-Th:hPDI3 and PTB7-Th:hPDI4 are 13.2, 13.1, 14.4 and 15.1 mA cm^−2^, respectively. These values agree well with measured values with a mismatch that is within 2% for all the devices. Spectra for the solar cells based on hPDI4 show an increase at 600 nm due to the intense absorption band for hPDI4 in this region. It is notable that all the EQE data consist of maximal transition bands around 550 nm that is mainly from the absorption of the acceptors. For those PTB7-Th-based solar cells, the highest EQE values are even beyond 70%. The important conclusion is that the photogenerated excitons from electron acceptors contribute greatly to the photocurrent in these solar cells.

### Exciton generation and charge transfer

Two advantages of these molecules over C_60_ and its derivatives are its significantly higher optical absorption cross-section for the molecules in the visible part of the solar spectrum and its easily tunable bandgap. The helical electron acceptors can thus complement low-bandgap electron donors for an efficient harvesting of solar radiation in a broad wavelength region. To establish this dual light-harvesting mechanism, we use TA spectroscopy to investigate exciton generation in the donor and acceptor, and charge transfer between the two materials.

**Figure 3:**
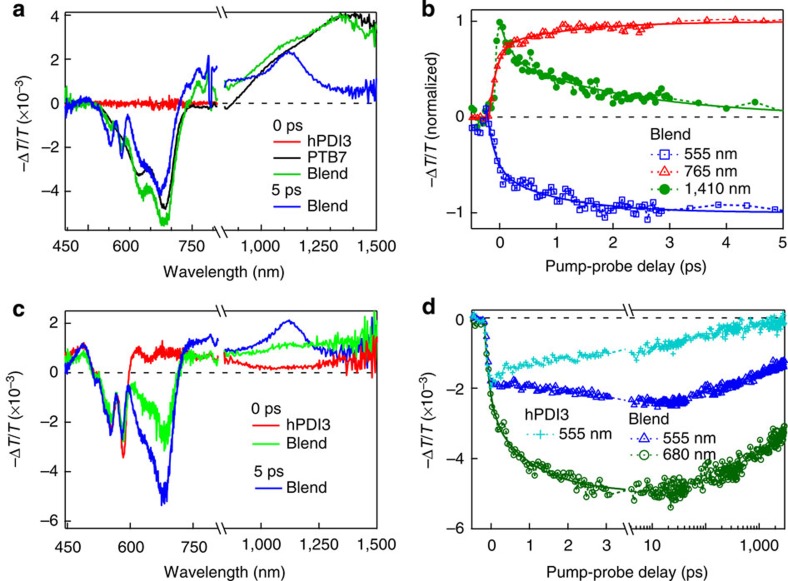
Spectral and temporal resolved ultrafast exciton dissociation. TA spectra for the films of neat hPDI3, neat PTB7 and their blended films, excited by 670 (**a**) and 415 nm (**c**). (**b**) Normalized dynamics in the blend at 555 nm (hPDI3 bleaching peak), polaron absorption at 765 nm and exciton ESA peak in PTB7 at 1,410 nm, on 670 nm excitation. (**d**) Dynamics at 555 nm for the neat hPDI3 and at 555 and 680 nm for the blended film on 415 nm excitation. The curve for neat hPDI3 (light blue) was scaled by a factor of 0.8. The pump density was ∼20 μJ cm^−2^ per pulse. Full lines in panels b and d are fits to the data.

Figure 3a compares TA spectra for neat films of hPDI3, PTB7 and the blend at 0 and 5 ps, respectively, on photoexcitation at 670 nm. At this wavelength, only PTB7 is excited, as confirmed by the absence of TA signal for hPDI3 (red). The spectrum from neat PTB7 (black) features ground-state bleaching (GB) peaks at 620 and 680 nm, and a broad excited state absorption (ESA) peaks at ∼1,400 nm; these features decay bi-exponentially with time constants of 3.2±0.3 and 50±4 ps (see Supplementary Fig. 18 and Supplementary Note 3). In the blend, this ESA feature decays rapidly, with two time constants of 0.12±0.03 and 1.4±0.2 ps (Fig. 3b), suggesting ultrafast electron transfer from photoexcited PTB7 to the electron acceptor, hPDI3. The ultrafast decay of the ESA at ∼1,410 nm is accompanied by a new ESA at ∼1,120 nm that is assigned to hole polaron absorption in PTB7. This assignment is consistent with previous spectroscopic measurement on chemical doped PTB7 that showed a polaron band at the same wavelength^30^. More evidences of this assignment are contained in the Supplementary Figs 19 and 20, and Supplementary Note 4.

The dynamics of exciton dissociation into charge carriers is characterized by two time scales, as shown by the biexponential fit (*τ*_1_=0.12±0.03 ps and *τ*_2_=1.4±0.2 ps) to the ESA signal probed at 1,410 nm (green dots and fit in Fig. 3b). This is more obvious in the ESA signal at ∼765 nm (red triangles in Fig. 3b) that is negligible for the neat PTB7 but appears in the blend. The new ESA in this region is assigned to the polaron absorption both in hPDI3 and PTB7. Bi-exponential fit (red curve, in Fig. 3b) to the dynamics at 765 nm yields time constants of *τ*_1_=0.12±0.03 ps (70% relative weight) and *τ*_2_=1.3±0.2 ps (30% relative weight). These time constants are identical to those obtained from fit-to-signal at 1,410 nm and both wavelengths probe the same dynamics: the decay of exciton in PTB7 (1,410 nm) and rise of polaron absorption (765 nm). We assign *τ*_1_ (0.12±0.03 ps) to the ultrafast exciton dissociation of PTB7 at the interface with electron acceptor hPDI3 (refs 16, 31, 32, 33, 34, 35) and *τ*_2_ (1.3±0.2 ps), to the diffusion of excitons in PTB7 towards interfaces before dissociation^36^. Further confirming the assignment of charge-transfer dynamics, we show that the appearance of GB from hPDI3 at 555 nm tracks the charge-transfer dynamics (blue squares and fit in Fig. 3b).

We now turn to complementary light harvesting by hPDI3 at its peak absorption wavelength of 415 nm (see Fig. 1d). For neat hPDI3, excitation at this wavelength leads to GB at 555 and 580 nm, as well as a broad ESA feature in the range of ∼600–1,000 nm (Fig. 3c). In the blend, excitation of hPDI3 leads to a rise of the bleaching of PTB7 at 680 nm. This bleaching signal grows with two time constants: *τ*_1_'=0.14±0.02 ps and *τ*_2_'=1.2±0.3 ps (green dots and fit in Fig. 3d). The former is assigned to ultrafast hole transfer from photoexcited hPDI3 to PTB7 and the latter to exciton diffusion time in hPDI3 towards interfaces before hole transfer. Exciton dissociation is also evident by the quenching of fluorescence from PTB7 on excitation of hPDI3 (Supplementary Fig. 21 and Supplementary Note 5). This interpretation is supported by the growth of ESA of hole polaron absorption in PTB7 at 1,120 nm (see Fig. 3c, blue spectrum at 5 ps). The charge separation process in the blend leads to an approximately two orders of magnitude increase in the time constant for the recovery of hPDI3 (probed at 555 nm, blue triangles in Fig. 3d) as compared with the same process in neat hPDI3 (light blue crosses in Fig. 3d). The similar longer carrier lifetime was also found in the blended film at different excitation wavelengths (Supplementary Fig. 18). The much longer carrier recombination times in the blend than those in neat films are attributed to slower bimolecular recombination of the spatially separated charges in the blend.

The TA results presented above establish that photoexcitation of either the donor or the acceptor contribute to charge-carrier generation. Similar to PC_71_BM-based BHJs^31^,^32^,^33^,^34^, ultrafast exciton generation from these acceptors in the blended film compensates photoexcitation of the donor, resulting in broad photoresponse over the visible light region. The prevalent use of fullerene has led to the proposal that the spherical shape of fullerene plays a unique role in providing connectivity and electronic delocalization essential for ultrafast charge separation^14^,^37^. However, our findings show that fullerene is not particularly special and the helical conjugated molecules can also lead to efficient charge separation in BHJs.

### Morphology characterization

It is well known that proper phase separation and interpenetrating networks of donor and acceptor domains are critical to charge separation and carrier transport. Many studies on the film morphology in polymer/fullerene solar cells suggest optimal morphology for device operation is to have aggregated domains with sizes on approximately tens of nanometres for both the donor and acceptor^13^,^38^. However, film morphology of non-fullerene solar cells has received less attention^15^,^16^,^17^,^18^. To study the morphology of our blend films, we first performed grazing-incident X-ray diffraction (GIXD) to understand molecular packing in our blend films. Neat films of both PTB7 and PTB7-Th are semicrystalline, as evident by the presence of their (100) reflections located at *q*=0.35 and *q*=0.30 Å^−1^, respectively, in the corresponding X-ray diffraction patterns in Supplementary Fig. 22a,b (refs 39, 40). The X-ray diffraction patterns also reveal (010) reflections in the out-of-plane direction between *q*=1.55 and *q*=1.85 Å^−1^. As the (010) reflections correspond to the direction of *π*-stacking, the intensity anisotropy corresponding to the (010) reflections indicate that both PTB7 and PTB7-Th preferentially adopt a face-on orientation. This assertion is consistent with the observation that the intensities of the (100) reflections of the polymer donors are each concentrated at the equator. The fact that the intensity distributions associated with the (100) and (010) reflections of PTB7-Th are sharper than those associated with the corresponding reflections of PTB7 indicates that the crystallites of PTB7-Th exhibit a higher degree of face-on orientation compared with PTB7. The GIXD pattern of hPDI3 in Supplementary Fig. 22c also exhibits a first-order reflection at *q*=0.35 Å^−1^. Given that the intensity distribution of this reflection does not vary substantially with azimuthal angle, crystallites of hPDI3 do not adopt any preferential orientation. Supplementary Fig. 22d,e exhibit the GIXD patterns of thin films comprising each of the polymer donor with hPDI3. The primary reflections of the polymer donors and hPDI3 overlap substantially. We are thus not able to make meaningful comparison based on the intensities in this *q*-range. Comparing the GIXD patterns of the blends at higher *q*-range with those of the constituent polymer donors does, however, reveal differences. We observe the (010) reflections in the GIXD patterns of the blends to be substantially weaker than those in the patterns of the polymer donor constituents. Further, the weak intensities associated with the (010) reflections in the GIXD patterns of the blends appear to be more isotropic with azimuthal angle. Collectively, these observations suggest the addition of hPDI3 to disrupt the crystallization of the polymer donor constituents and crystallites that form are less oriented compared with those in thin films comprising the polymer donor constituents alone.

**Figure 4:**
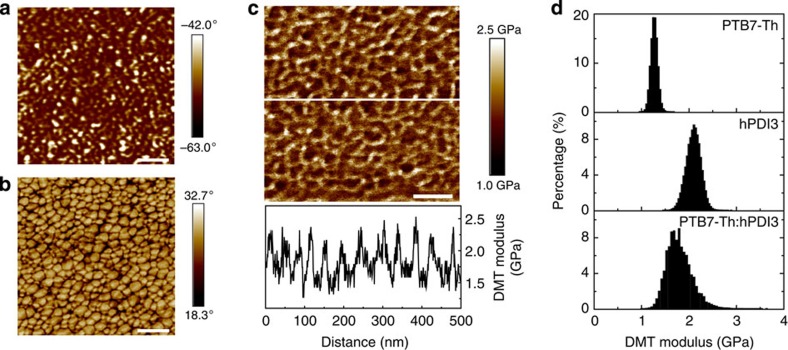
Film morphology of PTB7-Th: hPDI3 blend film. (**a**) Top surface phase image of BHJ thin film measured in tapping mode. (**b**) Internal phase image of blended thin film measured in tapping mode. (**c**) Internal DMT (Derjaguin, Muller, Toropov) modulus image of blended thin film measured in peak force quantitative nanomechanical (QNM) mode. Bottom graph is line-cut analysis of image. (**c**,**d**) DMT modulus of PTB7-Th and hPDI3 pure thin films and PTB7-Th:hPDI3 blend film. Scale bar, 100 nm (**a**–**c**).

We investigated the PTB7-Th:hPDI3-blended film morphology using both tapping mode and quantitative nanomechanical mode^41^ atomic force microscopy (AFM) (Fig. 4). Although the height image of the top film surface is very smooth (root-mean-squared (RMS) roughness of 0.58 nm), the phase image shows evidence of a distinct phase separation, with domain size of ∼10–20 nm (Fig. 4a and Supplementary Fig. 23). We used an oxygen plasma to remove ∼30 nm of material from the top surface to investigate the blend's internal morphology^41^,^42^,^43^. Here, the phase image clearly shows a continuous interpenetrating network with a feature size in the range of 20–40 nm (Fig. 4b)—a morphology clearly favourable for exciton dissociation and charge transport. Nanomechanical measurements show that the continuous network (dark regions in Fig. 4b) has a DMT (Derjaguin, Muller, Toropov) modulus of ∼2.2 GPa, which is similar to that of the pure film of hPDI3 (details about DMT model in Supplementary Note 6). However, the isolated embedded in the continuous network domains (bright regions in Fig. 4b) have a smaller DMT modulus (∼1.5 GPa), closer to that of a pure PTB7-Th film (Fig. 4c,d). These results suggest an active layer composed of an interpenetrating network of hPDI3-rich domains, embedded in PTB7-Th-rich matrix. Our blend films share very similar morphology to that of PTB7:PC_71_BM, which is considered an optimal morphology to enable efficient charge generation and transport in BHJ solar cells.^41^

## Conclusion

This study introduces a new class of highly efficient electron acceptors for organic BHJ solar cells, consisting of helical conjugated PDI oligomers. We have demonstrated solar cells with power conversion efficiencies comparable to previous reports from devices using fullerene derivatives. We believe the values reported here represent a lower limit for what is achievable, and that creating electron donor materials that are matched both structurally and electronically with these molecules is a clear path to further improvement.

## Methods

### Materials

hPDI3 and hPDI4 were synthesized according to a previously reported method^20^. PTB7 and PTB7-Th were purchased from 1-materal Inc. Synthesis of ZnO sol-gel precursor was described elsewhere^44^. Zinc acetate dihydrate, ethanolamine, 2-methoxyethanol, DIO and all of the solvents were purchased from Sigma Aldrich.

### Device fabrication

Prepatterned ITO-coated glass with a sheet resistance of ∼15 Ωsq^−1^ was cleaned with detergent and ultrasonicated in deionized water, acetone and isopropanol for 30 min, respectively. Subsequently, we treated the substrates by ultraviolet-ozone for 10 min. The prepared ZnO precursor was spin-cast onto the ITO substrate at 3,000 r.p.m. for 1 min, followed by annealing at 200 °C for 1 h in air, to form a thin film with approximate thickness of 20 nm. Active layers were prepared by spin-coating a mixed solution containing polymer and acceptor in chlorobenzene at a total concentration of 25 mg ml^−1^. The thickness of the prepared active layers is about 100 nm. Active layers were heated at 80 °C for 10 min in the nitrogen filled glove box to remove the residual solvent. Finally, a 7-nm MoO_3_ layer was deposited first and then a 100-nm Al electrode were subsequently deposited through a shadow mask by thermal evaporation under a vacuum about 1 × 10^−6^ torr.

### Characterization

The current density–voltage (*J*–*V*) curves were measured by a Keithley 2635A source measure unit. The photocurrent was measured under AM 1.5G illumination at 100 mW cm^−2^ under a Newport solar Simulator. A KG5-Si reference cell traceable to Newport was used to calibrate light intensity. Mismatch between integrated *J*_sc_ values from EQE with the AM 1.5G solar spectrum and the measured values is within 2%. Spectral mismatch factors (*M*) calculated according to a standard procedure reported elsewhere^45^ were applied to all the devices. The effective device area was defined as 6.25 mm^2^ by an aperture mask, except that a 4-mm^2^ aperture mask was used for the certified device at Newport Corporation. Average device parameters were obtained from at least 6 devices at each condition and at least 12 devices at the optimal condition for each donor–acceptor combination. EQE measurements were performed using a QE system from PV Measurements Inc. Absorption spectra were obtained on Shimadzu UV 1800 ultraviolet–visible.

### TA spectroscopy

To investigate charge transfer in the solar cells, we employed the TA spectroscopy. The pump laser light (∼100 fs pulse width) comes from an optical parametric amplifier pumped by a Ti:sapphire femtosecond regenerative amplifier (800 nm, 1 kHz rep-rate). The probe light is a white-light supercontinuum (450–900 and 850–1,600 nm for the visible and near-infrared range, respectively). The pump and probe beams overlapped under a small angle. The detection consists of a pair of high-resolution multichannel detector arrays coupled to a high-speed data acquisition system.

### Grazing-incident X-ray diffraction

GIXD experiments were conducted at the G1 station (11.8±0.1 keV) of the Cornell High Energy Synchrotron Source. The beam was chosen to be 0.05 mm tall and 1 mm wide. The width of each sample was 5 mm. The X-ray beam was aligned above the film's critical angle but below that of the substrate, at a 0.18° incident angle with the substrate. X-ray scattering was collected with a two-dimensional Pilatus 200 K detector, positioned 84.5 mm from the sample. All GIXD images have been background subtracted.

### Atomic force microscopy

AFM measurements were carried out in tapping mode and PeakForce quantitative nanomechanical mode on a Bruker Multi-Mode AFM at ambient conditions. A commercial silicon cantilever (RTESPA, MPP-11120-10, Bruker) was used in this study with a typical radius of curvature of ∼8 nm and a nominal spring constant of ∼40 Nm^−1^. Nanomechanical mapping was operated at constant peak force. The etching treatment was performed on the sample surface using oxygen plasma (Plasma Etch Inc., Model: PE-50) to etch out ∼30 nm from the top surface.

## Additional information

**How to cite this article:** Zhong, Y. *et al*. Molecular helices as electron acceptors in high-performance bulk heterojunction solar cells. *Nat. Commun*. 6:8242 doi: 10.1038/ncomms9242 (2015).

## Supplementary Material

Supplementary Information Supplementary Figures 1-24, Supplementary Tables 1-5, Supplementary Notes and Supplementary References.

## Figures and Tables

**Table 1:** Summary of device parameters of the best solar cells.

	***J***_**sc**_ **(mA** **cm**^**−2**^**) Highest/average**	***V***_**oc**_ **(V) Highest/average**	**FF (%) Highest/average**	**PCE (%) Highest/average**
PTB7:hPDI3	13.2/13.0±0.2	0.77/ 0.76±0.01	63/62±1	6.4/6.3±0.1
PTB7-Th:hPDI3	14.5/14.3±0.3	0.81/ 0.80±0.01	67/67±1	7.9/7.7±0.2
PTB7:hPDI4	12.9/12.7±0.3	0.79/0.78±0.01	64/63±1	6.5/6.4±0.1
PTB7-Th:hPDI4	15.2/15.0±0.2	0.80/0.80±0.01	68/68±1	8.3/8.1±0.2
PTB7-Th: hPDI4*	15.1	0.802	68.2	8.27

FF, fill factor; PCE, power conversion efficiency.

^*^Values were measured and certified by Newport Corporation.
