# Supplementary material for: Molecular helices as electron acceptors in high-performance bulk heterojunction solar cells
Source: Nat Commun. 2015 Sep 18;6:8242. doi: 10.1038/ncomms9242 (PMC4595599; doi:10.1038/ncomms9242)
Supplement: Supplementary Information — Supplementary Figures 1-24, Supplementary Tables 1-5, Supplementary Notes and Supplementary References. [file ncomms9242-s1.pdf]

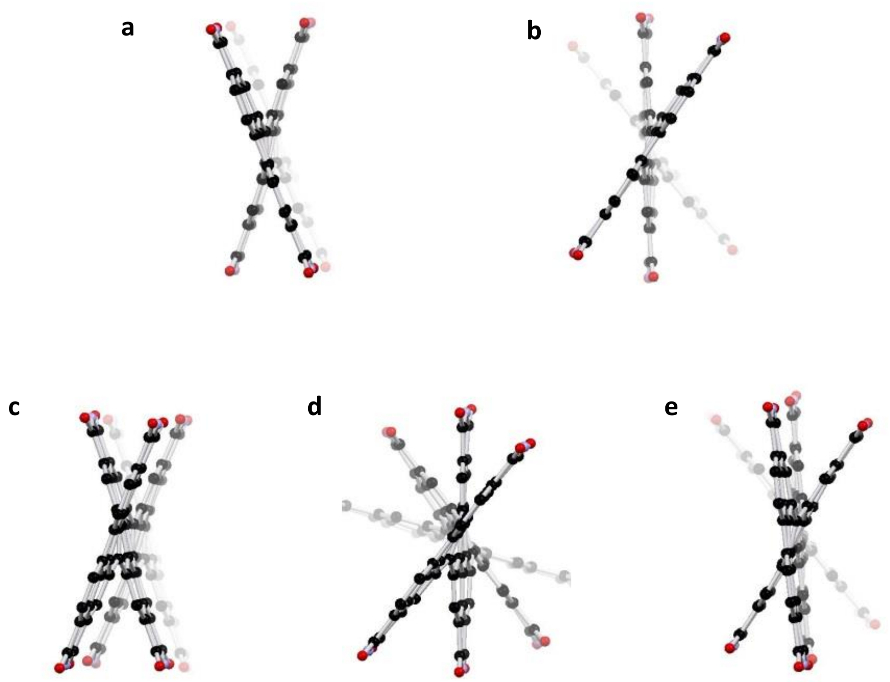

Supplementary Figure 1. Molecular conformation from DFT calculation. DFT models of (a-b) hPDI3 and (c-e) hPDI4. hPDI3 consists of two iso-energetic conformations, (a) "waggling" and (b) "helical". hPDI4 consists of three iso-energetic conformations, (c) "waggling", (d) "helical" and (e) "mixed". Hydrogens and alkyl side chains have been removed for clarity. Black = carbon; red = oxygen; blue = nitrogen.

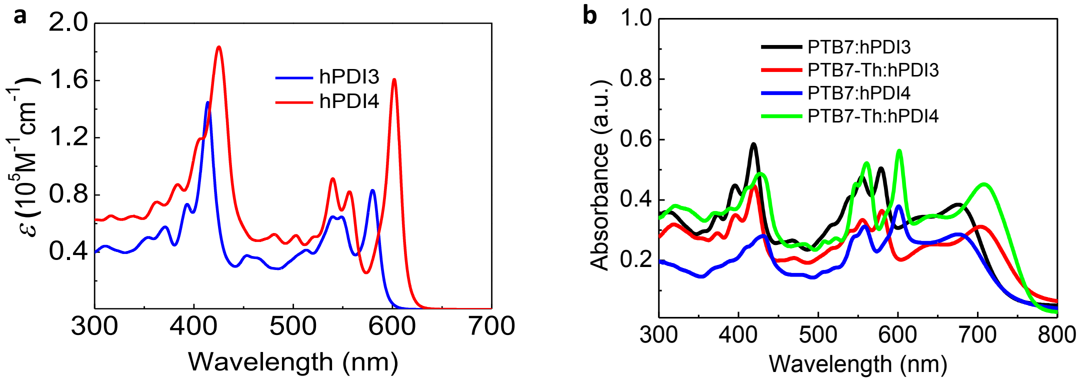

Supplementary Figure 2. Absorption spectra. (a) Solution absorption spectra of hPDI3 and hPDI4. (1×10^{-5} M concentration in dichloromethane with a path length l = 1 cm) (b) Film absorption spectra of PTB7:hPDI3 (1:1.5), PTB7:hPDI4 (1:1), PTB7-Th:hPDI3 (1:1.5) and PTB7-Th:hPDI4 (1:1).

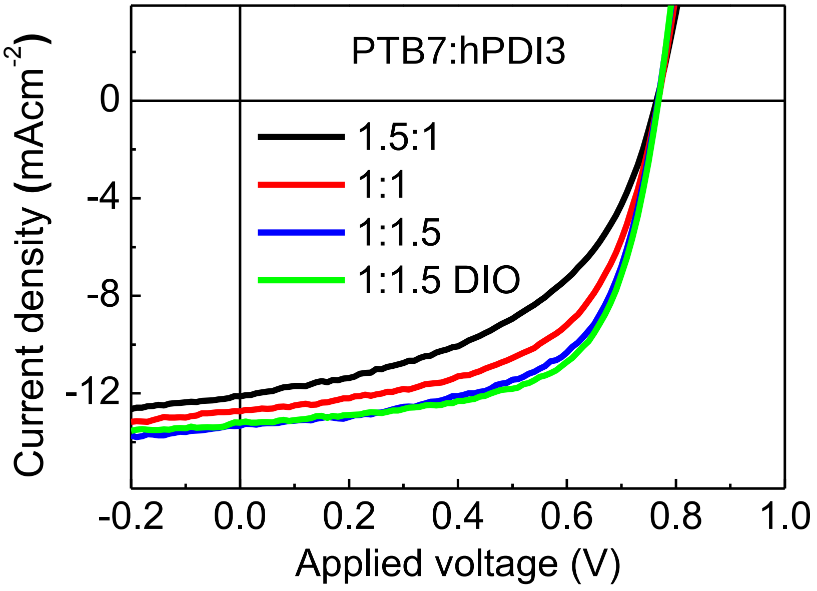

Supplementary Figure 3. Device performance of PTB7:hPDI3 solar cells. Current density versus voltage (*J*–*V*) characteristics of PTB7:hPDI3 solar cells with different blend ratios (PTB7 to hPDI3) and 1% v/v DIO.

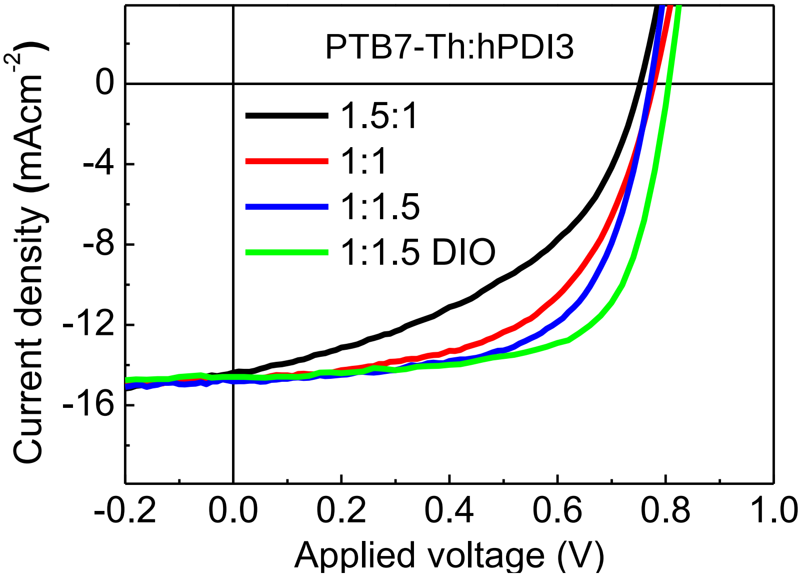

Supplementary Figure 4. Device performance of PTB7-Th:hPDI3 solar cells. Current density versus voltage (*J*–*V*) characteristics of PTB7-Th:hPDI3 solar cells with different blend ratios (PTB7-Th to hPDI3) and 1% v/v DIO.

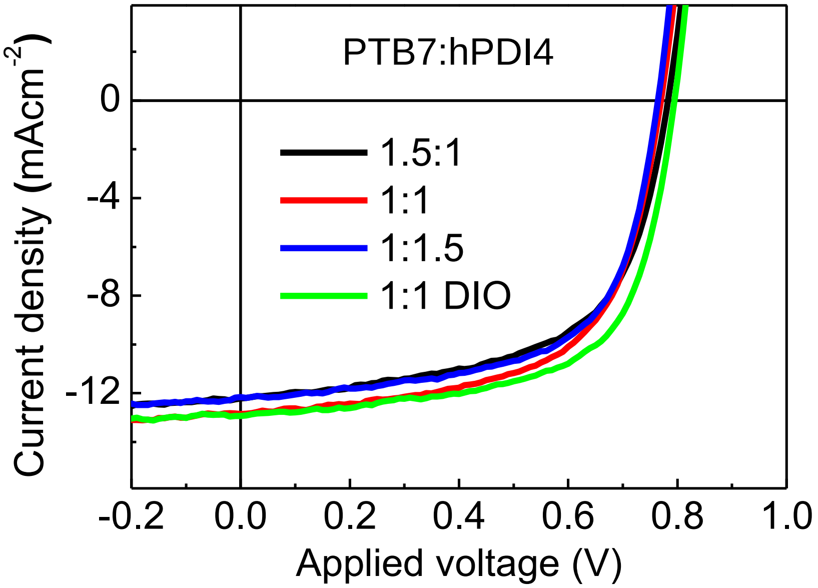

Supplementary Figure 5. Device performance of PTB7:hPDI4 solar cells. Current density versus voltage (*J*–*V*) characteristics of PTB7:hPDI4 solar cells with different blend ratios (PTB7 to hPDI4) and 1% v/v DIO.

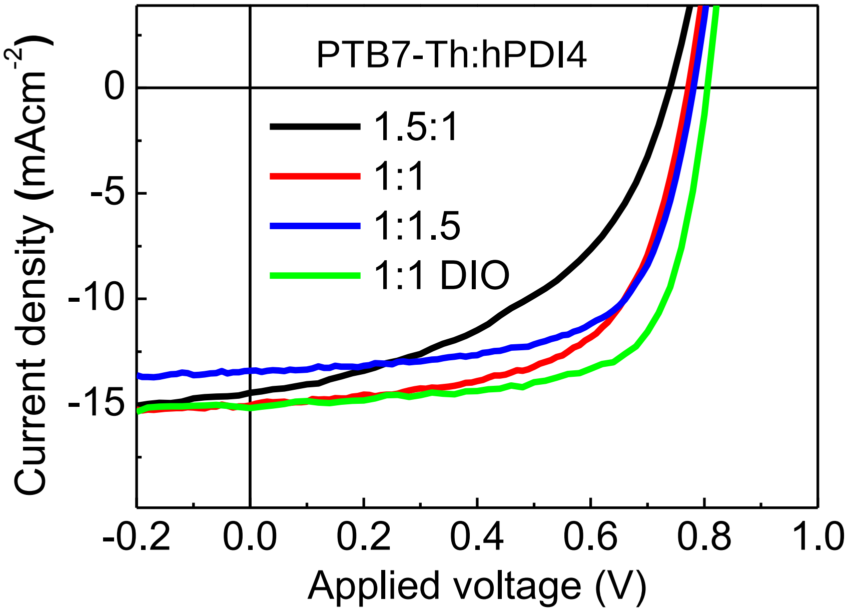

Supplementary Figure 6. Device performance of PTB7-Th:hPDI4 solar cells. Current density versus voltage (*J*–*V*) characteristics of PTB7-Th:hPDI4 solar cells with different blend ratios (PTB7-Th to hPDI4) and 1% v/v DIO.

Newport	ACCREDITED Calibration Cert. # 2893.01	Technology and Application Center **PV Lab** Newport Calibration Cert. # 1250

DUT S/N: 78P2
Newport Calibration #: 1250
Manufacturer: Professor Nuckolls Group
Material: OPV
Temperature Sensor: None
Environmental conditions at the time of calibration: Temperature: 24 ± 3 °C; Humidity: 33 ± 4 %

The above DUT has been tested using the following methods to meet the ISO 17025 Standard by the PV Lab at Newport Corporation. Quoted uncertainties are expanded using a coverage factor of $k = 2$ and expressed with an approximately 95% level of confidence. Measurement of total irradiance is traceable to the World Radiometric Reference (WRR) and all other measurements and uncertainties are traceable to either NIST or CNRC and the International System of Units (SI). The performance parameters reported in this certificate apply only at the time of the test, and do not imply future performance.

Efficency [%]	8.27 ± 0.18	V_oc [V]	0.8023 ± 0.0112	I_sc [A]	0.000596 ± 0.000011
P_max [mW]	0.326 ± 0.007	V_max [V]	0.6546 ± 0.0071	I_max [A]	0.000498 ± 0.000006
FF [%]	68.2 ± 1.0	Area [cm^2]	0.0394 ± 0.0001	M	0.989 ± 0.004

Methods:
I-V: ASTM E948-15 *Standard Test Method for Electrical Performance of Photovoltaic Cells Using Reference Cells Under Simulated Sunlight*
QE: ASTM E1021-15 *Standard Test Method for Spectral Responsivity Measurements of Photovoltaic Devices*

Standard Reporting Conditions:
Spectrum: AM1.5-G (ASTM G173-03/IEC 60904-3 ed. 2)
1000.0 W/m^2 at 25.0°C

Secondary Reference Cell:
Device S/N: PVM 284 KG5
Device Material: mono-Si
Window Material: KG5
Certification: National Renewable Energy Laboratory
A2LA accreditation certificate # 2236.01
ISO Tracking #: 1811
Certified short circuit current (I_{sc}) under standard reporting conditions (SRC): 33.803mA
Calibration due date: 11-Aug-15

Solar Simulator:
Spectrum: Newport Corporation filename *Sol3A_Spectroradiometer_Scan_0168.xls*
Total irradiance: 1000 W/m^2 based on I_{sc} of the above Secondary Reference Cell

Quantum Efficiency for DUT:
Newport Corporation filename *QE 1250 P2.log*
Spectral mismatch correction factor: M = 0.989 ± 0.004

DUT Calibration Procedures:
Newport Corporation document WI1 (EQE).docx
Newport Corporation document Area Measurement WI2 (Area).docx
Newport Corporation document WI3 (IV.Sweep).docx

Cal Cert V1.8	Issue Date: Jun 05, 2015	Page 2 of 2
	Reviewed and Approved by: Geoffrey Wicks	

Newport		Technology and Application Center<br>PV Lab<br><br>Calibration Number 1250

IV Curve Newport Cal Cert #1250

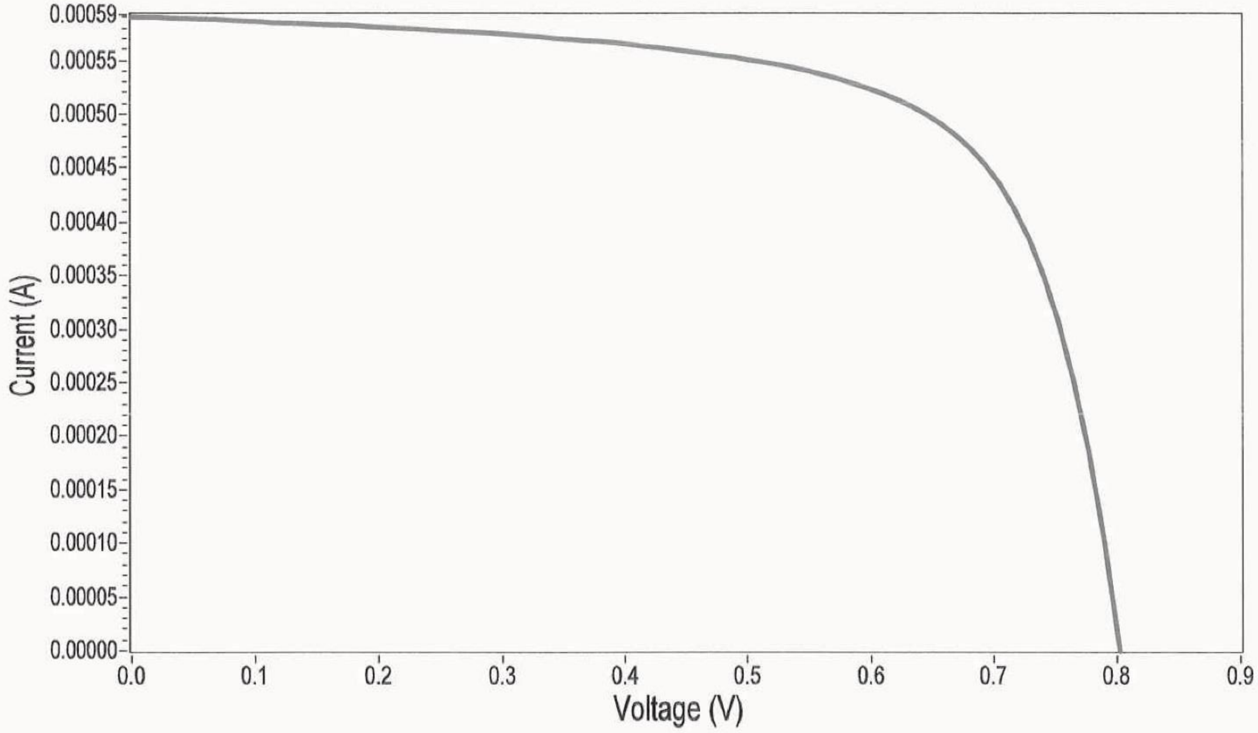

Cal Cert Data V1_2	Issue Date: Jun 05, 2015	Page 1of 4
	Reviewed and Approved by: Geoffrey Wicks	

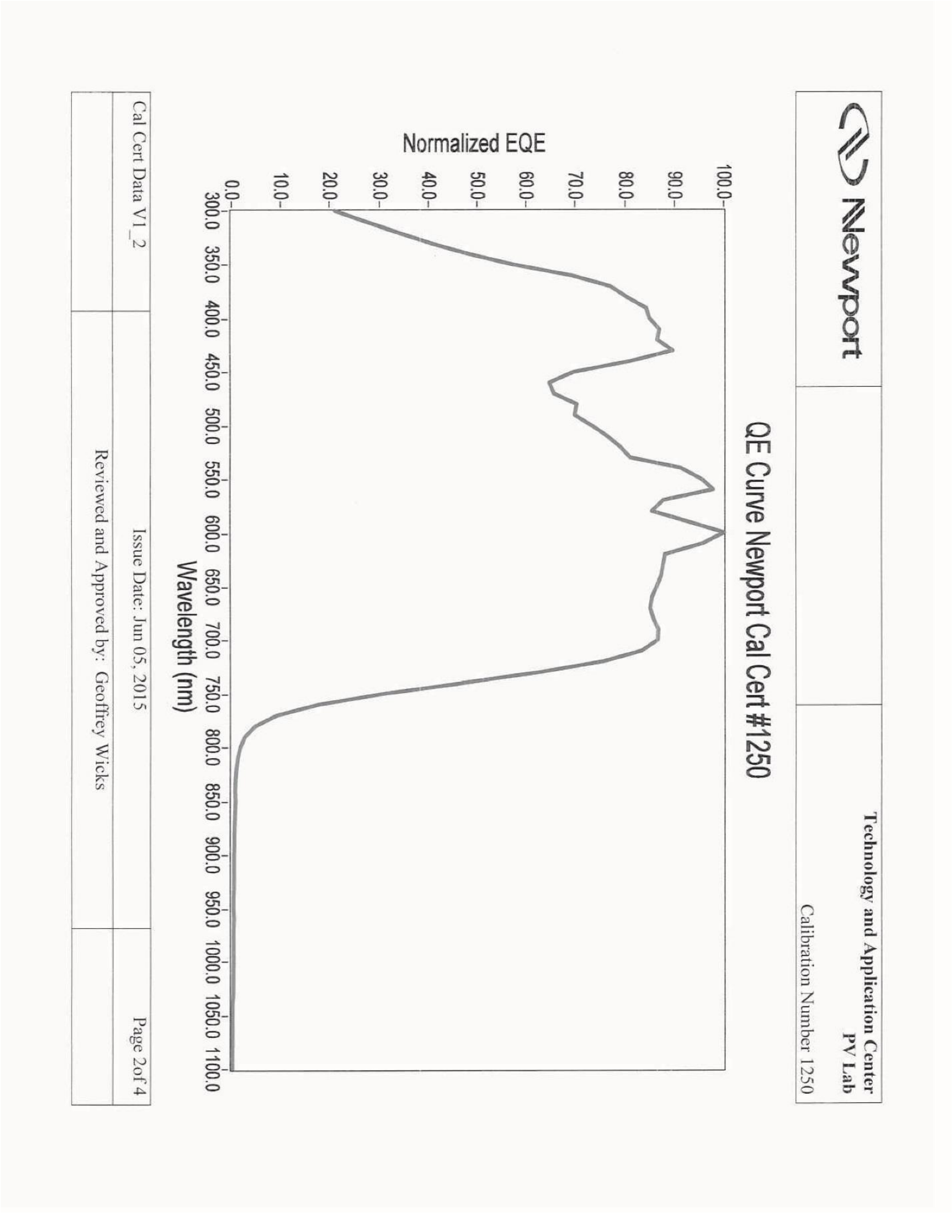

Supplementary Figure 7. Device characteristics of the PTB7-Th:hPDI4 solar cell tested by Newport Corporation.

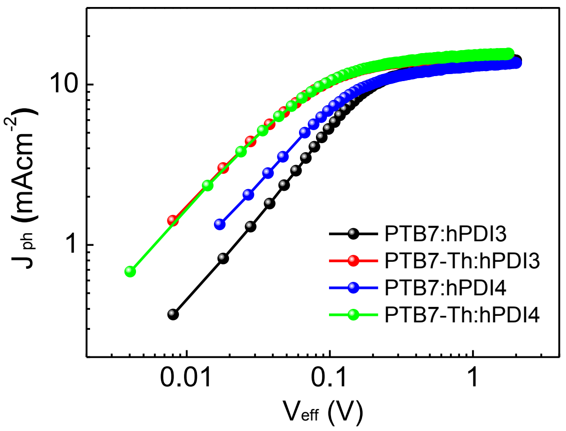

Supplementary Figure 8. Exciton generation rate. Photocurrent density (J_{ph}) versus effective voltage (V_{eff}) characteristics of PTB7:hPDI3, PTB7-Th:hPDI3, PTB7:hPDI4 and PTB7-Th:hPDI4 solar cells .

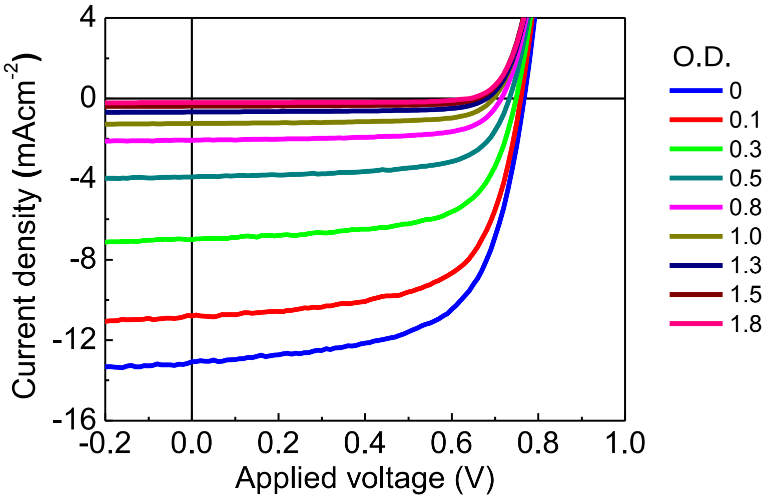

Supplementary Figure 9. Incident light intensity dependence of photocurrent on PTB7:hPDI3 solar cells. Current density versus voltage (*J*–*V*) characteristics for a PTB7:hPDI3 solar cell under AM 1.5 G illumination with a set of neutral density filters.

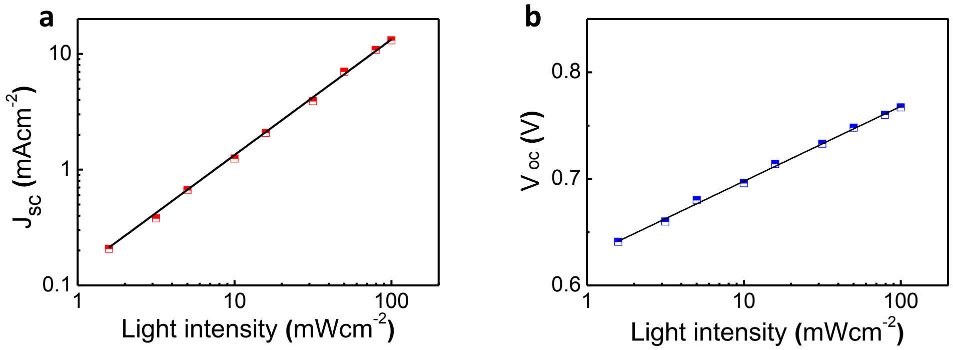

Supplementary Figure 10. Carrier recombination of PTB7:hPDI3 solar cells. (a) J_{sc} as a function of light intensity in a double-logarithmic scale from an optimized PTB7:hPDI3 device. The slope was calculated to be $k = 1.00 \pm 0.03$. (b) V_{oc} as a function of light intensity in a semi-logarithmic scale from an optimized PTB7:hPDI3 device. The slope was calculated to be $n = 1.17 \pm 0.02$ kT/e.

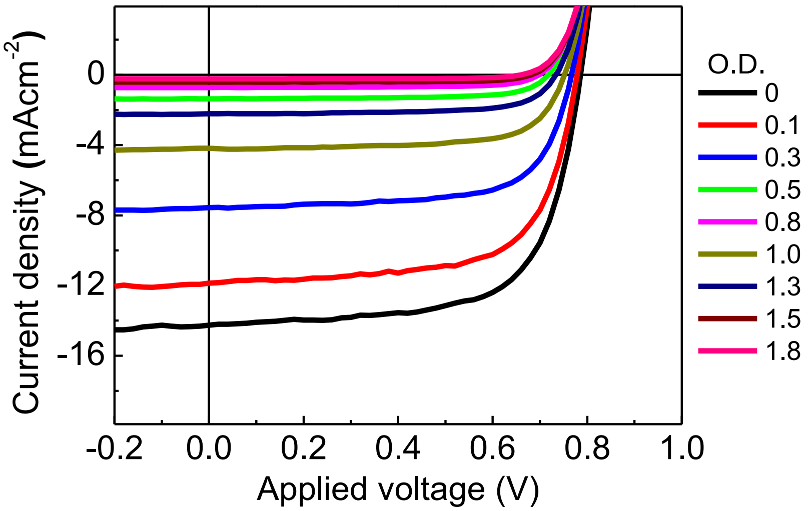

Supplementary Figure 11. Incident light intensity dependence of photocurrent on PTB7-Th:hPDI3 solar cells. Current density versus voltage (*J*–*V*) characteristics for a PTB7-Th:hPDI3 solar cell under AM 1.5 G illumination with a set of neutral density filters.

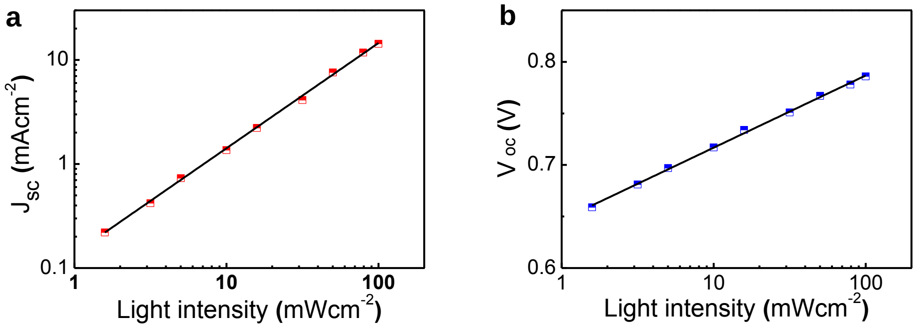

Supplementary Figure 12. Carrier recombination of PTB7-Th:hPDI3 solar cells. (a) J_{sc} as a function of light intensity in a double-logarithmic scale from an optimized PTB7-Th:hPDI3 device. The slope was calculated to be $k = 1.01 \pm 0.03$. (b) V_{oc} as a function of light intensity in a semi-logarithmic scale from an optimized PTB7-Th:hPDI3 device. The slope was calculated to be $n = 1.17 \pm 0.02$ kT/e.

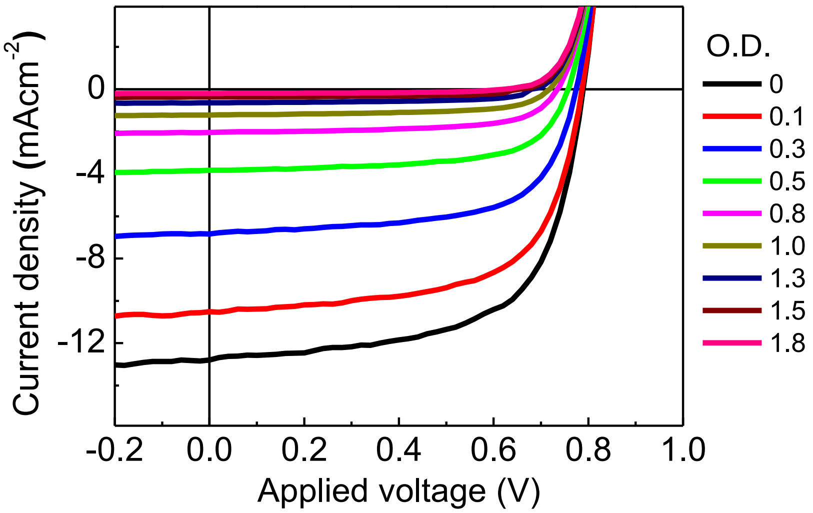

Supplementary Figure 13. Incident light intensity dependence of photocurrent on PTB7:hPDI4 solar cells. Current density versus voltage (*J*–*V*) characteristics for a PTB7:hPDI4 solar cell under AM 1.5 G illumination with a set of neutral density filters.

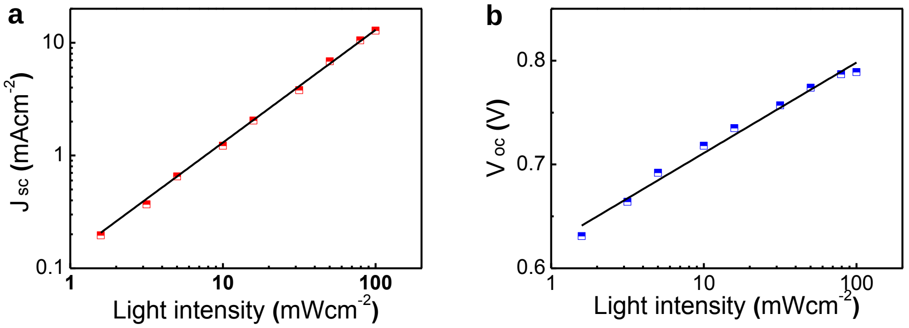

Supplementary Figure 14. Carrier recombination of PTB7:hPDI4 solar cells. (a) J_{sc} as a function of light intensity in a double-logarithmic scale from an optimized PTB7:hPDI4 device. The slope was calculated to be $k = 1.00 \pm 0.03$. (b) V_{oc} as a function of light intensity in a semi-logarithmic scale from an optimized PTB7:hPDI4 device. The slope was calculated to be $n = 1.46 \pm 0.06\ kT/e$.

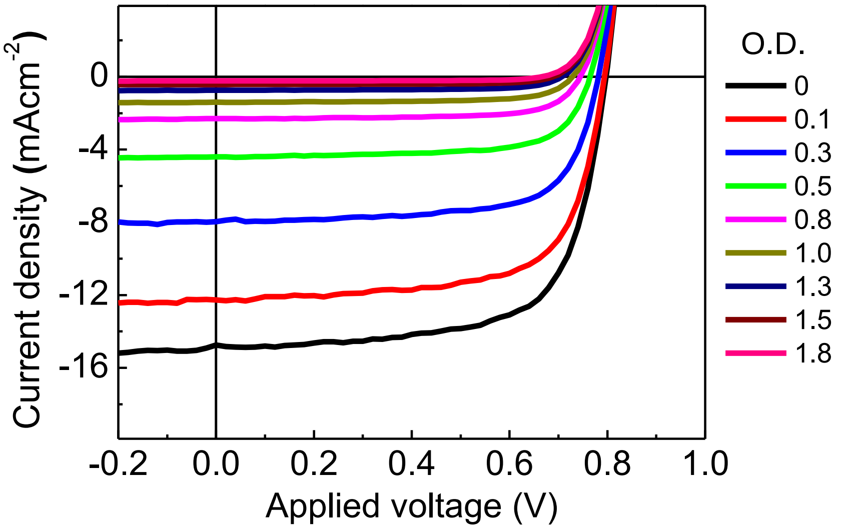

Supplementary Figure 15. Incident light intensity dependence of photocurrent on PTB7-Th:hPDI4 solar cells. Current density versus voltage (*J*–*V*) characteristics for a PTB7-Th:hPDI4 solar cell under AM 1.5 G illumination with a set of neutral density filters.

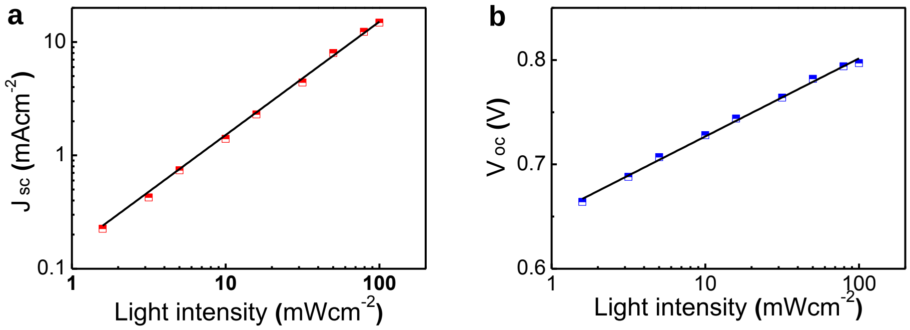

Supplementary Figure 16. Carrier recombination of PTB7-Th:hPDI4 solar cells. (a) J_{sc} as a function of light intensity in a double-logarithmic scale from an optimized PTB7-Th:hPDI4 device. The slope was calculated to be $k = 1.00 \pm 0.03$. (b) V_{oc} as a function of light intensity in a semi-logarithmic scale from an optimized PTB7-Th:hPDI4 device. The slope was calculated to be $n = 1.25 \pm 0.03\ kT/e$.

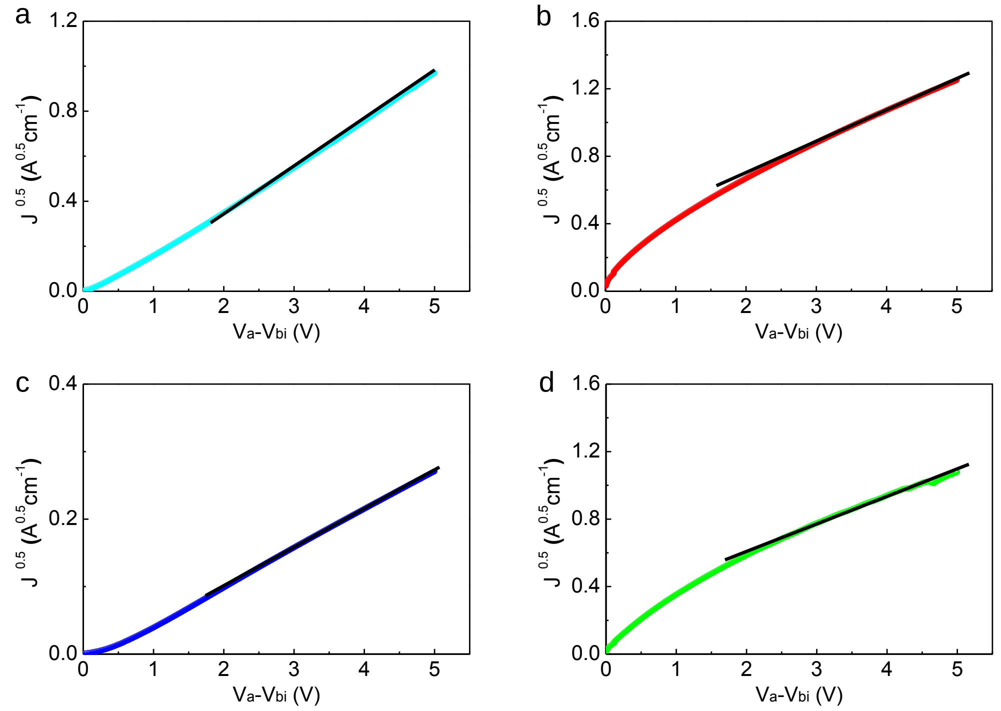

Supplementary Figure 17. Carrier mobility. $J^{0.5}$ vs. V plots for (a) an electron-only device, (b) a hole-only device from PTB7-Th:hPDI3 blend and (c) an electron-only device, (d) a hole-only device from PTB7-Th:hPDI4 blend.

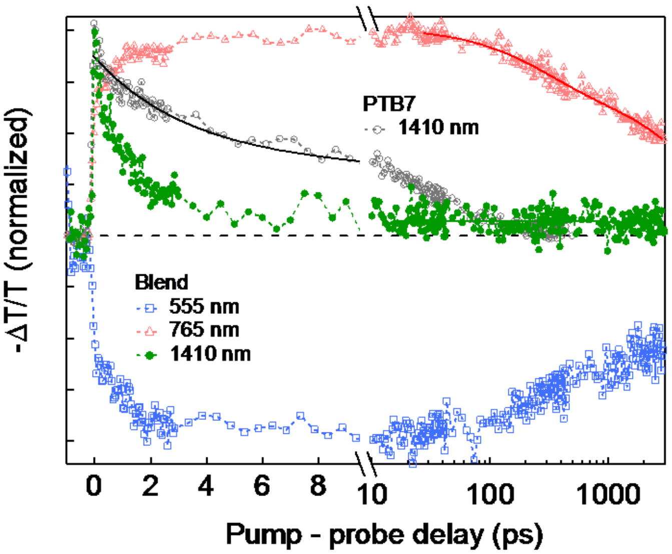

Supplementary Figure 18. **Exciton/carrier dynamics.** Exciton/carrier dynamics upon 670 nm excitation. Exciton dynamics at 1410 nm in the neat PTB7 film (grey) and the PTB7:hPDI3 blended film (green). Charge (polaron) dynamics at 555 (blue) and 765 (red) nm for the blended film.

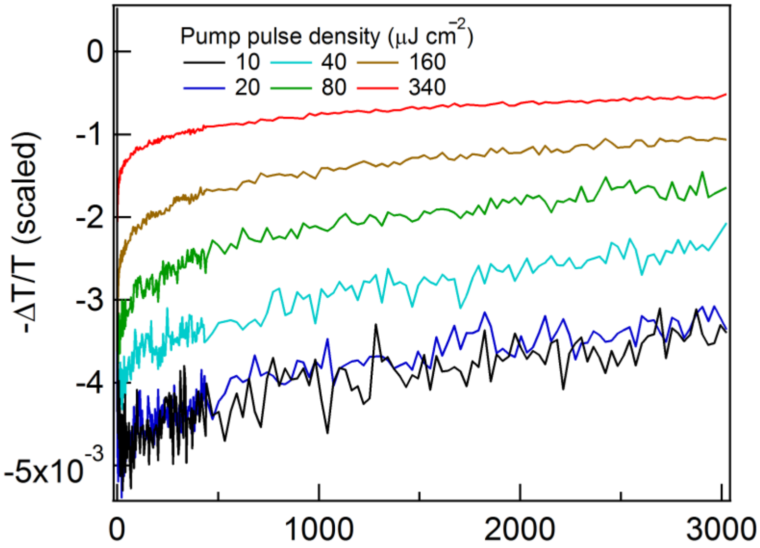

Supplementary Figure 19. Dynamics at 680 nm (PTB7 bleaching) upon 415 nm excitation at various pump intensities for the blended film. The curves were normalized for the pump intensities. The dynamics are similar for the pump intensities of 10 and 20 μJcm^{-2} indicating of negligible non-linear effect at these pump intensities. At pump intensities larger than 40 μJcm^{-2} the fast component appears due to carrier-carrier annihilation. The initial bleaching values is lower for a higher pump intensities (from 40 μJcm^{-2}) due to the annihilation that takes place earlier than exciton diffusion time.

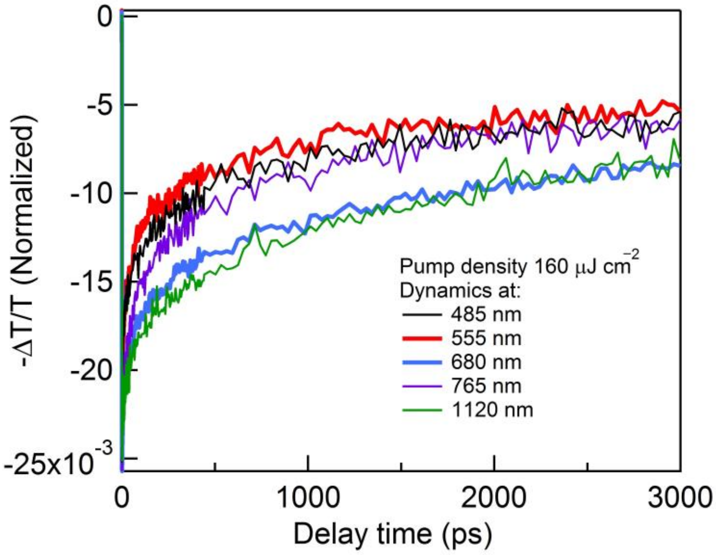

Supplementary Figure 20. **Normalized dynamics at various probe wavelengths for the blend at a high pump density (160 μJcm^{-2}).** Excitation wavelength was 415 nm. Dynamics at 555 (red) and 680 (blue) nm represent the bleaching dynamics for hPDI3 and PTB7 in the blend, respectively. Dynamics at 485 nm, 555 nm, 765 nm and 1120 nm was scaled by a factor of -3.2, 1.5, -3.15 and -1.9, respectively, for normalization.

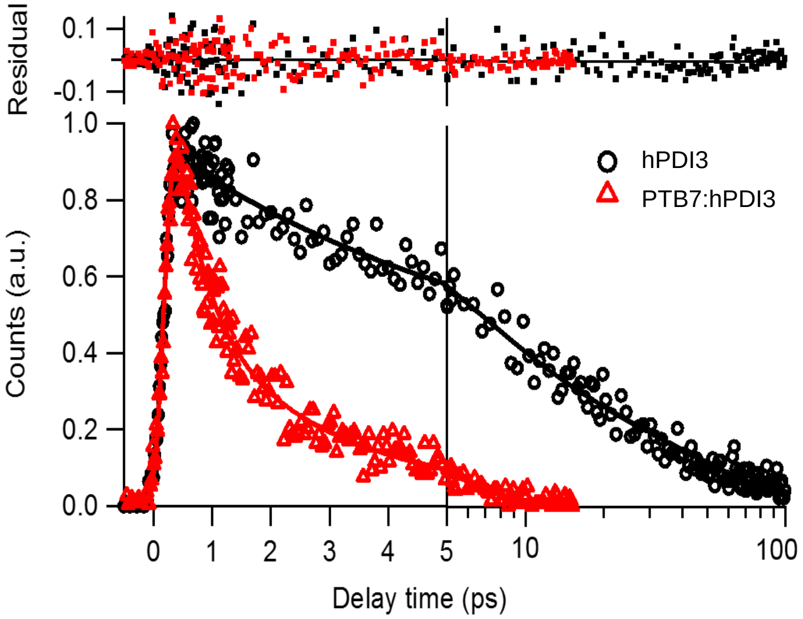

Supplementary Figure 21. Upconverted fluorescence decay kinetics for hPDI3 (black circles) and blended film (red triangles). The decay kinetics are fitted by bi-exponential decay function. The excitation wavelength is 508 nm and the upconverted PL is centered at 580 nm.

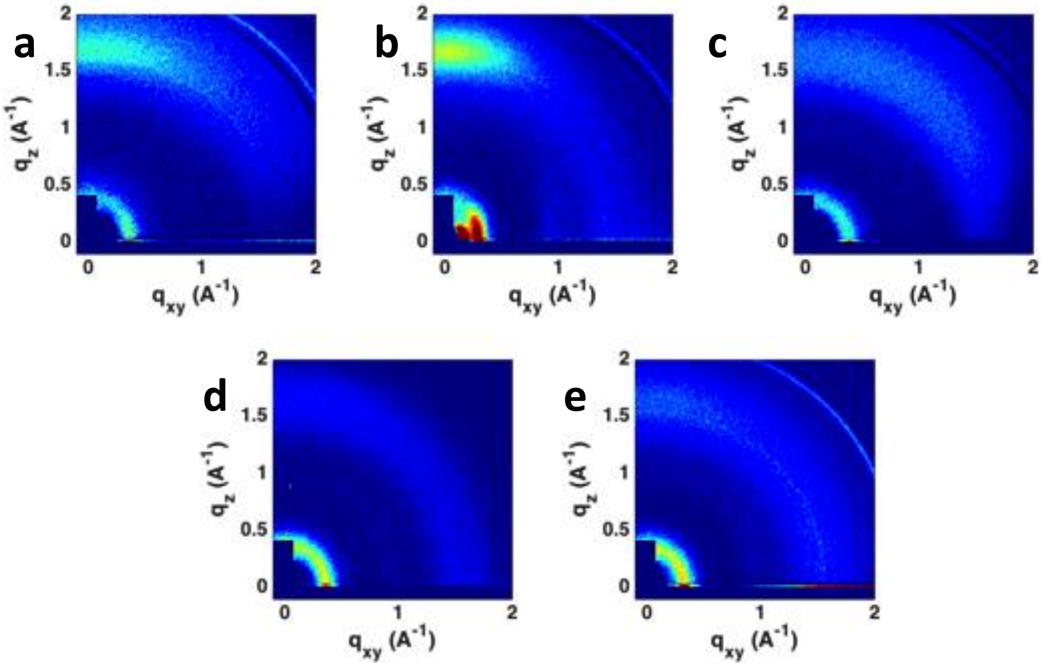

Supplementary Figure 22. Grazing incident X-ray diffraction. GIXD images of (a) PTB7, (b) PTB7-Th, (c) hPDI3, (d) PTB7:hPDI3 with 1% DIO and (e) PTB7-Th:hPDI3 with 1% DIO on ZnO coated ITO substrates. Blend films of PTB7:hPDI3 and PTB7-Th:hPDI3 were prepared at same conditions as used in optimal solar cells.

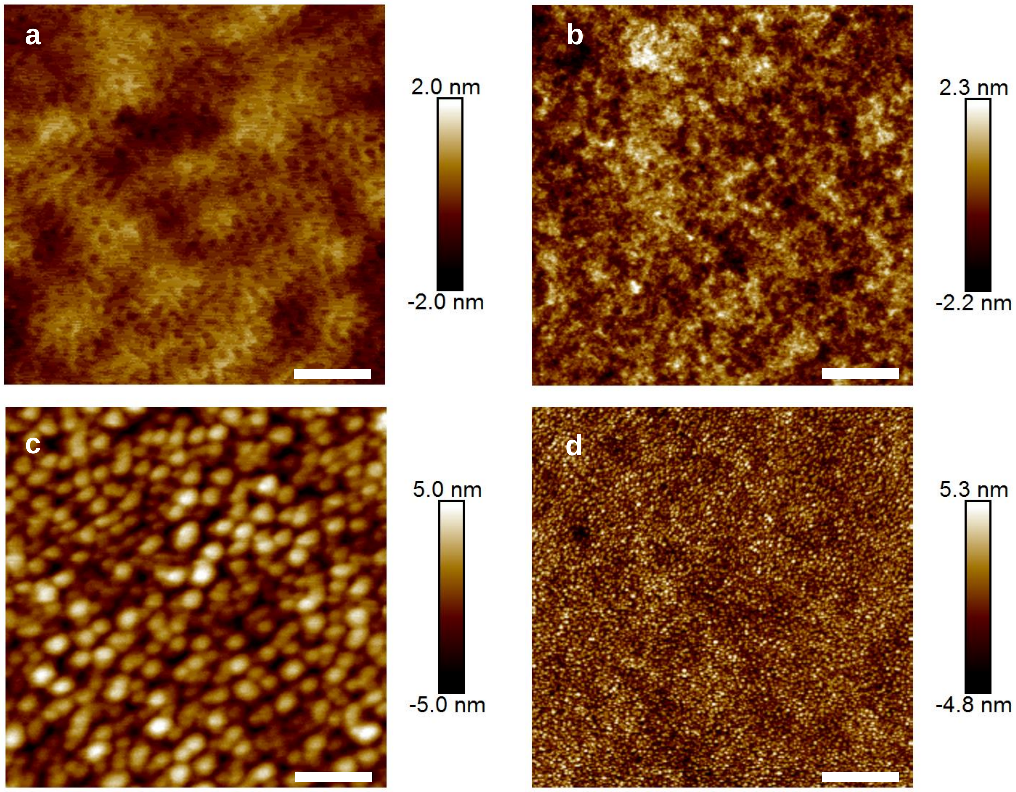

Supplementary Figure 23. Atomic force microscopy. Top surface (a and b) and internal (c and d) morphology of PTB7-Th:hPDI3 blend films at a mass ratio of 1:1.5 processed from chlorobenzene solutions with 1v/v% DIO. All the films were annealed at 80 ^{o}C for 10 min in the nitrogen-filled glove box. Height images of (a and c) 500 nm × 500nm scan, and of (b and d) 3um × 3um scan. The scale bar is 100 nm in (a) and (c), and 600 nm in (b) and (d), respectively.

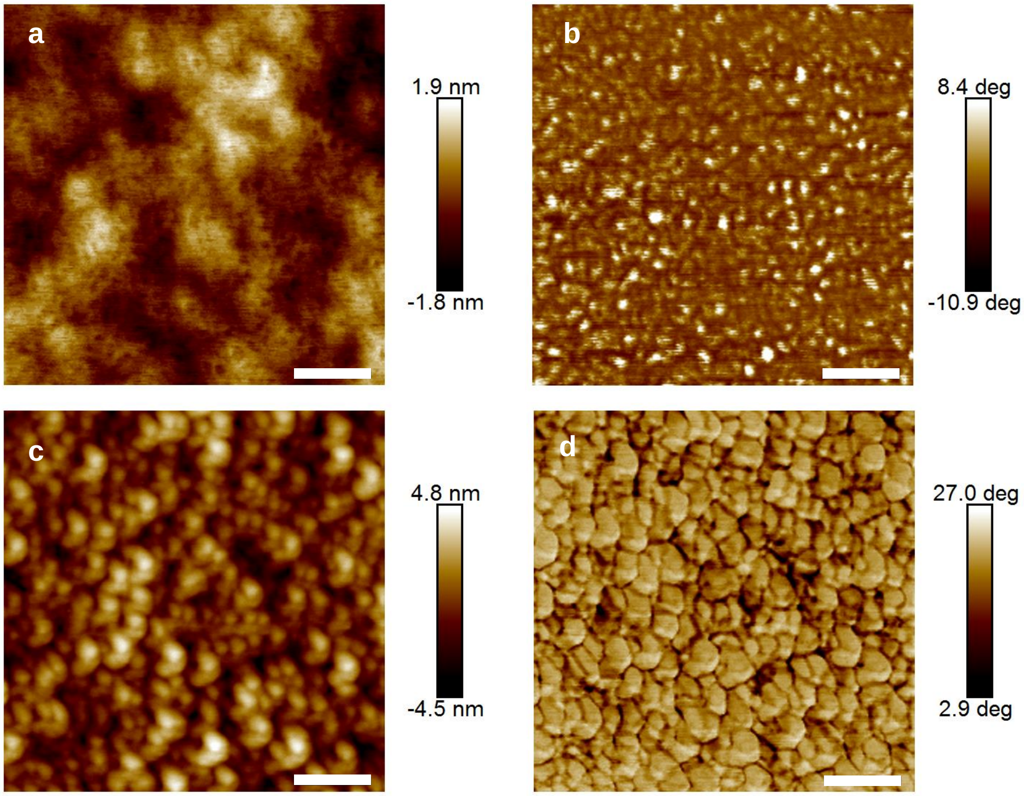

Supplementary Figure 24. Atomic force microscopy. Top surface (a and b) and internal (c and d) morphology of PTB7:hPDI3 blend films at a mass ratio of 1:1.5 processed from chlorobenzene solutions with 1v/v% DIO. All the films were annealed at 80 °C for 10 min in the nitrogen-filled glove box. (a) Height image of top surface, (b) phase image of top surface, (c) height image of internal morphology and (d) phase image of internal morphology. The scale bar is 100 nm.

Supplementary Tables

Supplementary Table 1. Summary of device parameters of best PTB7:hPDI3 solar cells with different conditions.

PTB7:hPDI3	J_{sc} (mAcm^{-2}) Highest/Avg.	V_{oc} (V) Highest/Avg.	FF (%) Highest/Avg.	PCE (%) Highest/Avg.
1.5:1	12.1/12.0 ± 0.2	0.76/0.75 ± 0.01	49/48 ± 1	4.5/4.4 ± 0.1
1:1	12.7/ 12.5 ± 0.2	0.77/0.76 ± 0.01	56/55 ± 1	5.5/5.4 ± 0.1
1:1.5	13.3/ 13.1 ± 0.2	0.77/0.76 ± 0.01	61/60 ± 1	6.2/6.1 ± 0.1
1:1.5 w/ 1% DIO	13.2/13.0 ± 0.2	0.77/ 0.76 ± 0.01	63/62 ± 1	6.4/6.3 ± 0.1

Supplementary Table 2. Summary of device parameters of best PTB7-Th:hPDI3 solar cells with different conditions.

PTB7-Th:hPDI3	J_{sc} (mAcm^{-2}) Highest/Avg.	V_{oc} (V) Highest/Avg.	FF (%) Highest/Avg.	PCE (%) Highest/Avg.
1.5:1	14.4/14.1 ± 0.3	0.75/0.75 ± 0.01	47/ 47 ± 1	5.1/4.9 ± 0.2
1:1	14.7/14.5 ± 0.2	0.78/0.77 ± 0.01	56/55 ± 1	6.4/6.3 ± 0.1
1:1.5	14.8/14.5 ± 0.3	0.77/0.77 ± 0.01	62 /62 ± 1	7.1/6.9 ± 0.2
1:1.5 w/ 1% DIO	14.5/14.3 ± 0.3	0.81/ 0.80 ± 0.01	67/67 ± 1	7.9/7.7 ± 0.2

Supplementary Table 3. Summary of device parameters of best PTB7:hPDI4 solar cells with different conditions.

PTB7:hPDI4	J_{sc} (mAcm^{-2}) Highest/Avg.	V_{oc} (V) Highest/Avg.	FF (%) Highest/Avg.	PCE (%) Highest/Avg.
1.5:1	12.2/12.0 ± 0.3	0.78/0.77 ± 0.01	60/59 ± 1	5.7/5.4 ± 0.3
1:1	13.1/12.9 ± 0.2	0.77/0.76 ± 0.01	62/61 ± 1	6.2/6.1 ± 0.1
1:1.5	12.2/12.1 ± 0.2	0.76/0.76 ± 0.01	62/62 ± 1	5.8/5.6 ± 0.2
1:1 w/ 1% DIO	12.9/12.7 ± 0.3	0.79/0.78 ± 0.01	64/63 ± 1	6.5/6.4 ± 0.1

Supplementary Table 4. Summary of device parameters of best PTB7-Th:hPDI4 solar cells with different conditions.

PTB7-Th:hPDI4	J_{sc} (mAcm^{-2})	V_{oc} (V)	FF (%)	PCE (%)
1.5:1	14.5/14.5 ± 0.2	0.74/0.74 ± 0.01	46/45 ± 1	4.9/4.7 ± 0.2
1:1	15.0/14.9 ± 0.3	0.77/0.77 ± 0.01	61/61 ± 1	7.1/7.0 ± 0.1
1:1.5	13.4/13.2 ± 0.2	0.78/0.78 ± 0.01	65/65 ± 1	6.8/6.6 ± 0.2
1:1 w/ 1% DIO	15.2/15.0 ± 0.2	0.80/0.80 ± 0.01	68/68 ± 1	8.3/8.1 ± 0.2

Supplementary Table 5. Summary of saturated photocurrent, exciton generation rate and charge collection probability at J_{sc} condition of best solar cells.

	**PTB7:hPDI3**	**PTB7:hPDI4**	**PTB7-Th:hPDI3**	**PTB7-Th:hPDI4**
$J_{ph,sat}$ **(mAcm^{-2})**	14.1	13.6	15.0	15.6
G_{max} **(cm^{-3}s^{-1})**	8.8 x 10^{21}	8.5 x 10^{21}	9.4 x 10^{21}	9.7 x 10^{21}
P_c **(J_{sc})**	0.94	0.97	0.97	0.97

Supplementary Notes

Supplementary Note 1. DFT calculation methods. All quantum chemical calculations were performed using Jaguar, version 8.2, Schrodinger, Inc., New York, NY, 2013. (See A. D. Bochevarov, E. Harder, T. F. Hughes, J. R. Greenwood, D. A. Braden, D. M. Philipp, D. Rinaldo, M. D. Halls, J. Zhang, R. A. Friesner, "Jaguar: A HighPerformance Quantum Chemistry Software Program with Strengths in Life and Materials Sciences", Int. J. Quantum Chem., 2013, 113(18), 2110-2142). All geometries were optimized using the B3LYP functional and the 6-31G** basis set. In the case of each molecule, the energies of the different conformations were compared by performing additional calculations at the several optimized geometries using the larger cc-pvtz basis set. We observe no significant energy differences between the different local minima in either case.

To simplify the calculations, we replaced the $C_{11}H_{23}$ side chain attached to each nitrogen atom with a single H. The influence of alkyl N-substituents on the electronic structures is negligible due to the nodes of frontier orbitals at the imide nitrogens.[1, 2] The twisted conformation of the molecules is a consequence of repulsion between the two C −H bonds on the

inner bay position of adjacent perylene diimide (PDI) units. DFT calculations show that hPDI3 has two isoenergetic conformers while hPDI4 has three. Multiple conformations originate from the relative helicity at each of the fusing points of adjacent PDI units.

Supplementary Note 2. Carrier mobility measurement. Carrier mobility was measured by space charge limit current (SCLC) method. Hole-only devices were fabricated with a device configuration of ITO/PEDOT:PSS/PTB7-Th:hPDI3 or hPDI4/MoO_3/Al. Electron-only devices were fabricated with a device configuration of ITO/ZnO/ PTB7-Th:hPDI3 or hPDI4/Al. The mobility was determined by fitting the dark current to the model of a single carrier SCLC, described by the equation:

$$J = \frac{9}{8}\varepsilon_0\varepsilon_r\mu\frac{V^2}{d^3} \quad (1)$$

where J is the current density, μ is the carrier mobility under zero field, ε_0 is the permittivity of free space, ε_r is the material relative permittivity (assumed to be 3 here), d is the active layer thickness (100 nm for PTB7-Th:hPDI3 and 110nm for PTB7-Th:hPDI4), and V is the effective voltage. The effective voltage can be obtained by subtracting the built-in voltage (V_{bi}) from the applied voltage (V_a),[3] $V=V_a - V_{bi}$. V_{bi} is 0 V for hole-only devices and 0.23 V for electron-only devices. The carrier mobility can be calculated from the slope of the $J^{0.5}$~V curves. The mobility values were averaged from 4 devices for each one.

Supplementary Note 3. Exciton/carrier dynamics. Supplementary Figure 18 shows exciton/carrier dynamics upon 670 nm excitation. The excited state absorption (ESA) exciton dynamics at 1410 nm (grey curve) for the neat PTB7 film decays bi-exponentially with two time constants of 3.25 ± 0.2 ps and 56 ± 2 ps. In the blend, the dynamics at this wavelength (green)

decays with one order of magnitude shorter lifetimes in comparison to that of the neat film indicating of ultrafast electron transfer from PTB7 to hPDI3. While the exciton feature for PTB7 in the blends rapidly decay, the dynamics at 555 and 765 nm decay much slower with time constants of 275 ± 40 ps and 6.5 ± 0.6 ns. The slower decay is attributed to bimolecular recombination between electron in hPDI3 and hole in PTB7.

Supplementary Note 4. Assignment of excited state absorption. Supplementary Figure 20 shows normalized dynamics at different probe wavelengths upon 415 nm excitation at 160 μJcm^{-2} excitation intensity for the blended film. At this high pump intensity, the carrier-carrier annihilation takes place in PTB7 and hPDI3 domains. The annihilation rate depends on the charge density and mobility in each domain. The dynamics at 555 (red) and 680 (blue) nm represent to the bleaching dynamics of hPDI3 and PTB7 in the blend, respectively. These bleaching dynamics decay differently because carrier-carrier annihilation rates are different in these two domains. While the ESA dynamics at 485 nm (black) is similar to that at 555 nm, the ESA dynamics at 1120 nm (green) is similar to 1120 nm dynamics. These results suggest that the ESA at 485 nm comes from polaron (electron) absorption in hPDI3 while the ESA at 1120 nm comes from polaron (hole) absorption in PTB7. The ESA dynamics at 765 nm (purple) decays as a mixing of bleaching dynamics in hPDI3 and PTB7 indicates that at this probe wavelength region there is an overlap of ESAs for polarons in both donor and acceptor.

Supplementary Note 5. Ultrafast fluorescence upconversion. The fluorescence upconversion experiment was based on a Ti: Sapphire regenerative amplifier laser system with average power of 1.1 W, lasing wavelength of 805 nm, repetition rate of 250 kHz and pulse

duration of about 100 fs (Coherent Mira seeded RegA). 85% of fundamental pulse was sent to pump Coherent optical parametric amplifier, from which 508 nm excitation beam was generated. The fluorescence emitted from the sample was collected and focused into a 0.5 mm thick type I BBO crystal by a pair of off-axis parabolic silver mirror and spatially overlapped with the rest (15%) of fundamental beam as gate pulse. The upconverted emission was sent through a monochromator (corresponding to emission wavelength at 580 nm, the peak of hPDI3 emission) and detected by photomultiplier tube.

Supplementary Note 6. Atomic force microscopy. AFM measurements were carried out in tapping mode and PeakForce QNM (Quantitative NanoMechanics) mode on a Bruker Multi-Mode AFM at ambient conditions. A commercial silicon cantilever (RTESPA, MPP-11120-10, Bruker) was used in this study with a typical radius of curvature of ~8 nm and a nominal spring constant of ~40 Nm^{-1}. Nanomechanical mapping was operated at constant peak force. The etching treatment was performed on the sample surface using O_2 plasma (Plasma Etch Inc. Model: PE-50) to etch out ~30 nm from the top surface. The reduced Young's Modulus, E^*, is obtained by fitting the retract curve using the Derjaguin, Muller, Toropov (DMT) model[4] given by

$$F_{\mathrm{tip}} = \frac{4}{3}E^*\sqrt{Rd^3} + F_{\mathrm{adh}} \tag{2}$$

where F_{tip} is the force on the tip, F_{adh} is the adhesion force, R is the tip end radius and d is the tip sample separation. The reduced modulus is related to the sample modulus by the following equation:

$$E^* = \left[\frac{1-v_{\mathrm{t}}^2}{E_{\mathrm{t}}} + \frac{1-v_{\mathrm{s}}^2}{E_{\mathrm{s}}}\right]^{-1} \tag{3}$$

where v_{t} and E_{t} are the Poisson's ratio and Young's modulus of the tip and v_{s} and E_{s} are the

Poisson's ratio and Young's modulus of the sample. We assume that the tip modulus, E_t, is much larger than the sample modulus, E_s, and can be approximated as infinite and calculate the sample modulus using the sample Poisson's Ratio. Poisson's ratio generally ranges between about 0.2 and 0.5 (perfectly incompressible) giving a difference between the reduced modulus and the sample modulus between 4% and 25%. Because the sample's Poisson's ratio is not generally known, here we report reduced Young's modulus as many publications do.[5]

Supplementary References

1. Sang Kwon, L. et al. Electrochemistry, Spectroscopy and Electrogenerated Chemiluminescence of Perylene, Terrylene, and Quaterrylene Diimides in Aprotic Solution. *J. Am. Chem. Soc.* **121**, 3513–3520 (1999).
2. Wurthner, F. Perylene bisimide dyes as versatile building blocks for functional supramolecular architectures. *Chem. Commun.*, 1564-1579 (2004).
3. Malliaras, G.G., Salem, J.R., Brock, P.J. & Scott, C. Electrical characteristics and efficiency of single-layer organic light-emitting diodes. *Phys. Rev. B* **58**, R13411-R13414 (1998).
4. Derjaguin, B.V., Muller, V.M. & Toporov, Y.P. Effect of contact deformations on the adhesion of particles. *J. Colloid Interface Sci.* **53**, 314-326 (1975).
5. examples can be found in Bruker PeakPorce QNM user guide, www.bruker.com/products/surface-analysis/atomic-force-microscopy/modes/modes/imaging-modes/peakforce-qnm.html. (2015)
